# The treatment of intravascular leiomyomatosis with different clinical manifestations: Two case reports

**DOI:** 10.1097/MD.0000000000044406

**Published:** 2025-09-05

**Authors:** Yiyang Shi, Jiaxi Wang, Xuelu Jiang, Chen Zhang

**Affiliations:** a Department of the First School of Clinical Medicine, Zhejiang Chinese Medical University, Hangzhou, Zhejiang, People’s Republic of China; b Department of Gynecology and Obstetrics, The First Affiliated Hospital of Zhejiang Chinese Medical University, Hangzhou, Zhejiang, China.

**Keywords:** differential diagnosis, imaging diagnosis, intravascular leiomyomatosis, Qiu empirical formula, surgeries

## Abstract

**Rationale:**

Intravascular/intravenous leiomyomatosis (IVL) is a rare benign smooth muscle cell tumor with malignant biological behavior. The early diagnosis of IVL is challenging, and the range of treatment options is extensive.

**Patient concerns:**

Herein, we present 2 cases of IVL that present markedly different clinical presentations. These cases underscore the importance of vigilance in the diagnosis of IVL. A further objective of this study is to demonstrate the similarities and differences in treatment modalities. Patient 1 was registered for lower abdominal discomfort and a palpable pelvic mass, with a high preoperative suspicion of IVL. Patient 2 was characterized by severe vaginal bleeding during menstruation, accompanied by a palpable uterine mass, and an initial diagnosis of uterine adenomyosis or fibroids, with suspicion of IVL.

**Diagnoses:**

Both patients’ diagnoses were confirmed as IVL by histopathology.

**Interventions:**

Removal of the uterine lesion combined with bilateral salpingo-oophorectomy was performed in the former case, whereas total hysterectomy and bilateral salpingo-oophorectomy were performed in the latter case. Both were treated postoperatively with Qiu empirical formula, a traditional Chinese medicine herbal decoction.

**Outcomes:**

No recurrence was observed in either patient.

**Lessons:**

In the 2 cases examined in this study, following initial evaluation by imaging and complete resection of the lesion by surgical treatment initially, the patients demonstrated a more favorable prognosis following the application of herbal preparations in the long-term postoperative follow-up period. Our work provides additional information for clinicians to further study and better understand specific types of leiomyosarcoma.

## 1. Introduction

Intravascular/intravenous leiomyomatosis (IVL) is a rare form of uterine fibroids (UFs) that is characterized by the proliferation of benign smooth muscle within the myometrial vasculature and the subsequent extension into the extrauterine venous system, including the adjacent pelvic veins, iliac veins, and inferior vena cava, and even potentially into the heart and pulmonary blood vessels.^[1]^ Although IVL is rare, its true incidence remains uncertain, but it has been estimated to affect approximately 0.1% of women undergoing surgery for uterine leiomyomas, predominantly in women of reproductive or perimenopausal age.^[2]^ The etiology and pathogenesis of IVL remain uncertain, and the clinical manifestations are diverse, ranging from nonspecific symptoms such as lower abdominal discomfort, abnormal uterine bleeding, pelvic mass, or lower extremity edema to severe, life-threatening cardiac symptoms in advanced stages.^[3,4]^ The lesion typically extends from the myometrial or uterine venous system to the pelvic veins, iliac veins, inferior vena cava, and in advanced cases, may reach the right atrium, right ventricle, or even pulmonary arteries.^[5]^ Complete surgical resection remains the mainstay of treatment, typically involving total hysterectomy combined with careful excision of the intravascular tumor to reduce recurrence.^[6]^ Given the challenges in early diagnosis and the diversity of therapeutic strategies for IVL, we present 2 representative cases to highlight its variable clinical manifestations and management approaches.

## 2. Case presentation

### 2.1. Patient 1

A 45-year-old female patient, gravida 5 para 2, was admitted to the gynecology department due to lower abdominal pain during menstruation and 10 days before menstruation, which had started 1 year previously. The severity of menstrual pain had increased over time, reaching a more acute state in recent months. A physical examination conducted 13 years prior to admission revealed the presence of multiple UFs. At that time, the largest UF was approximately 6 cm in size. A Laparoscopic myomectomy was performed, and postoperative pathology confirmed the presence of UFs. Subsequent regular ultrasound examinations postoperatively indicated recurrence of the fibroids. However, no specific treatment was administered. No significant medical history was reported, nor was there any family history of UFs.

**Figure 1.**
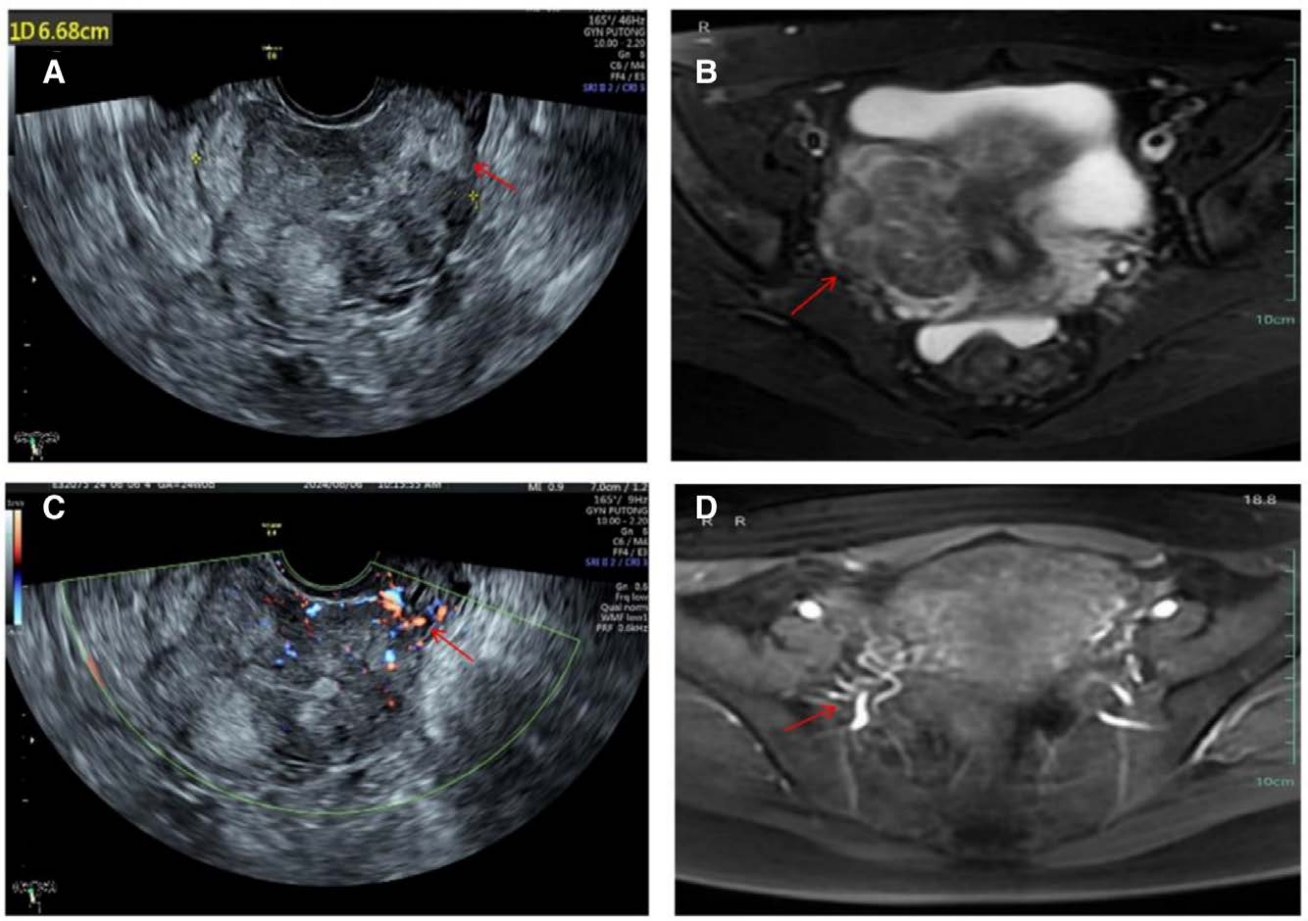
The clinical and pathological features of Patient 1. (A and B) Pelvic ultrasonography and MRI shows the location and morphology of the tumor (approximately 6 cm in diameter). (C and D) CDFI shows a rich blood supply with visible vessel penetration within the mass. The contrast agent shows thickening of the contrast-enhanced blood vessels. CDFI = color Doppler flow imaging, MRI = magnetic resonance imaging.

Physical examination revealed that the uterus had enlarged to the size of a 3-month pregnancy and exhibited an uneven surface. Pelvic ultrasonography at the outpatient clinic revealed a hypoechoic, sausage-like mass on the right wall of the isthmus of the uterus, measuring approximately 6.1 × 4.1 × 6.7 cm, and extending to the right parametrium. In addition, ultrasonography identified a small area of mixed hypoechoic and hyperechoic signals measuring 5.3 × 4.9 × 4.1 cm, a hypoechoic nodule (1.5 × 1.0 cm) in the myometrium of the left posterior wall, and a hyperechoic nodule (0.6 × 0.3 cm) in the uterine cavity (Fig. 1). To further evaluate these findings and clarify the extent of the lesion and its possible intravascular involvement, contrast-enhanced magnetic resonance imaging was subsequently performed. magnetic resonance imaging revealed that the sausage-like mass demonstrated continuous, cord-like extension along the expected course of the uterine and pelvic veins, without a clear boundary from adjacent vascular structures, suggesting the possibility of intravascular growth rather than simple myometrial involvement. The results of the blood test taken after admission revealed a cancer antigen 125 (CA125) level of 64.8 U/mL (normal value: 0–47 U/mL) and an estradiol level of 652.45 pmol/L on the 20th day of the menstrual cycle. Given the slightly elevated CA125 level and the presence of a large pelvic mass with suspected extrauterine extension, chest computed tomography (CT) was performed to exclude thoracic involvement, including possible tumor thrombus extension to the pulmonary vasculature or lung metastases, which may occur in advanced cases of intravascular leiomyomatosis. No obvious abnormalities were identified on chest CT, deep veins of the lower extremities, or electrocardiogram. In view of the imaging features indicating a vascular-like growth pattern, the lesion’s extension trajectory, and the absence of a capsule typical of leiomyoma, a preliminary diagnosis of IVL was made.

Subsequent surgical intervention included laparoscopic removal of the uterine lesions and both fallopian tubes. Intraoperatively, it was observed that the uterus had significantly enlarged to the size of the 3rd trimester of pregnancy. A tumor-like bulge measuring approximately 7 × 5 cm in size was identified in the right isthmus, near the peritoneum of the posteriorly tilted bladder, and was oriented toward the right broad ligament. After the plasma membrane was incised, the leiomyoma was observed to extend downward and backward along the uterine arteries and veins. Concurrently, the right round ligament, the anterior and posterior lobes of the broad ligament, and the right peritoneum were meticulously stripped. The source of the uterine artery was meticulously clamped, and the fibroid was meticulously excised from the vessel, the right isthmus, and the right neck wall. The specimen was then sutured. Additionally, visible fibroids, adenomyosis, and multiple polypoid tumor-like lesions in the uterine cavity were meticulously excised and sutured.

**Figure 2.**
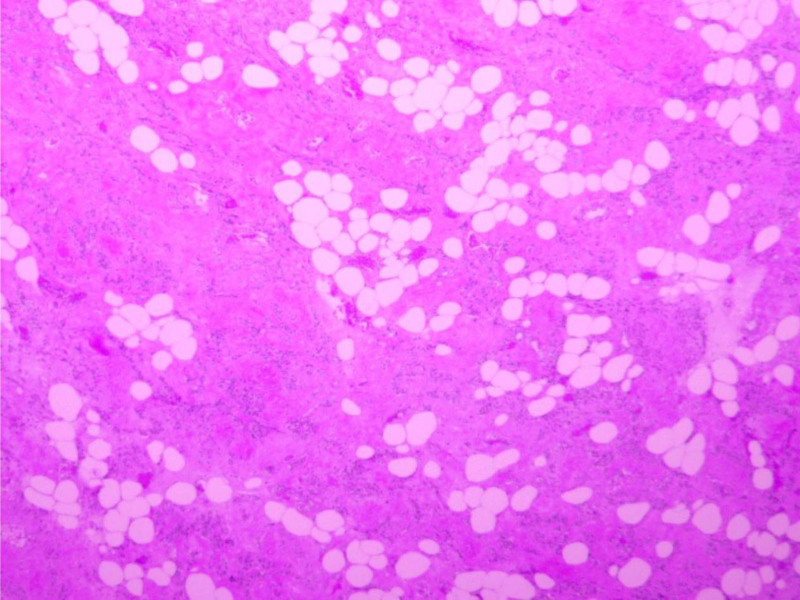
Intravascular tumors filling the venous lumen were stained with hematoxylin and eosin (H&E). The typical spindle cells were arranged in an interlaced pattern, there was no obvious atypia, and the vessel wall had smooth muscle components.

**Figure 3.**
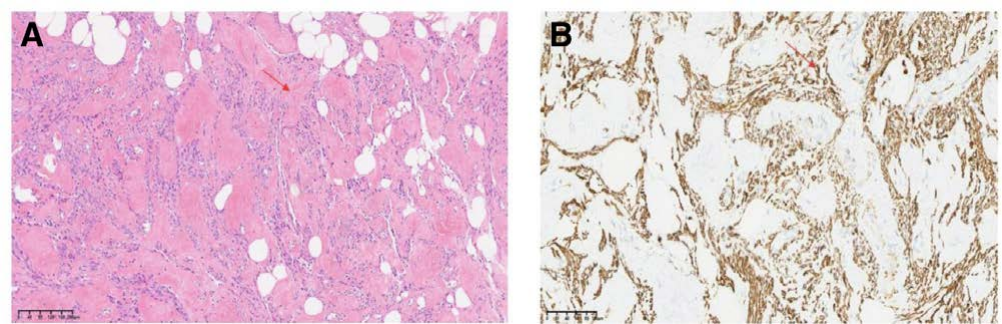
(A) Intravascular tumors filling the venous lumen were stained with hematoxylin and eosin (H&E). The typical spindle cells were arranged in an interlaced pattern, there was no obvious atypia, and the vessel wall had smooth muscle components. (B) The tumor is positive for Desmin marker, confirming the component of smooth muscle in IVL. IVL = intravascular/intravenous leiomyomatosis.

Microscopic examination revealed a smooth muscle tumor component, which was partially located in the vasculature, with hyaline degeneration visible in the interstitium (Fig. 2). Immunohistochemical analysis revealed positive expression of Desmin, D2-40 (lymphatic vessels), CD34 (vasculature), and Ki-67 (< 5%+), and the pathological findings corroborated the diagnosis of IVL (Fig. 3). Concomitant gynecological conditions included adenomyosis and endometrial polyps.

After surgery, the patient’s follow-up chest CT and echocardiographic examinations revealed no significant abnormalities. The patient was discharged from the hospital 5 days after undergoing the surgical procedure and exhibited no complications. Subsequent monthly follow-ups and long-term use of Qiu empirical formula^[7]^ have not revealed any signs of recurrence.

### 2.2. Patient 2

A 44-year-old female patient, gravida 2 para 1, was diagnosed with adenomyosis and a submucosal UF 6 years prior, based on transvaginal ultrasound and pelvic examination findings. She gradually developed menorrhagia and dysmenorrhea, for which she underwent 2 hysteroscopic surgeries – 5 years ago and 1 year ago – to remove the submucosal fibroids and resect most of the adenomyotic tissue in order to alleviate bleeding. However, in the past month, she experienced a recurrence of heavy menstrual bleeding, necessitating readmission to the gynecology department. Repeat ultrasound revealed uterine adenomyosis, most of which was removed by hysteroscopy. She had a cesarean section 24 years prior and hyperthyroidism for 1 year. She had no other relevant medical history.

**Figure 4.**
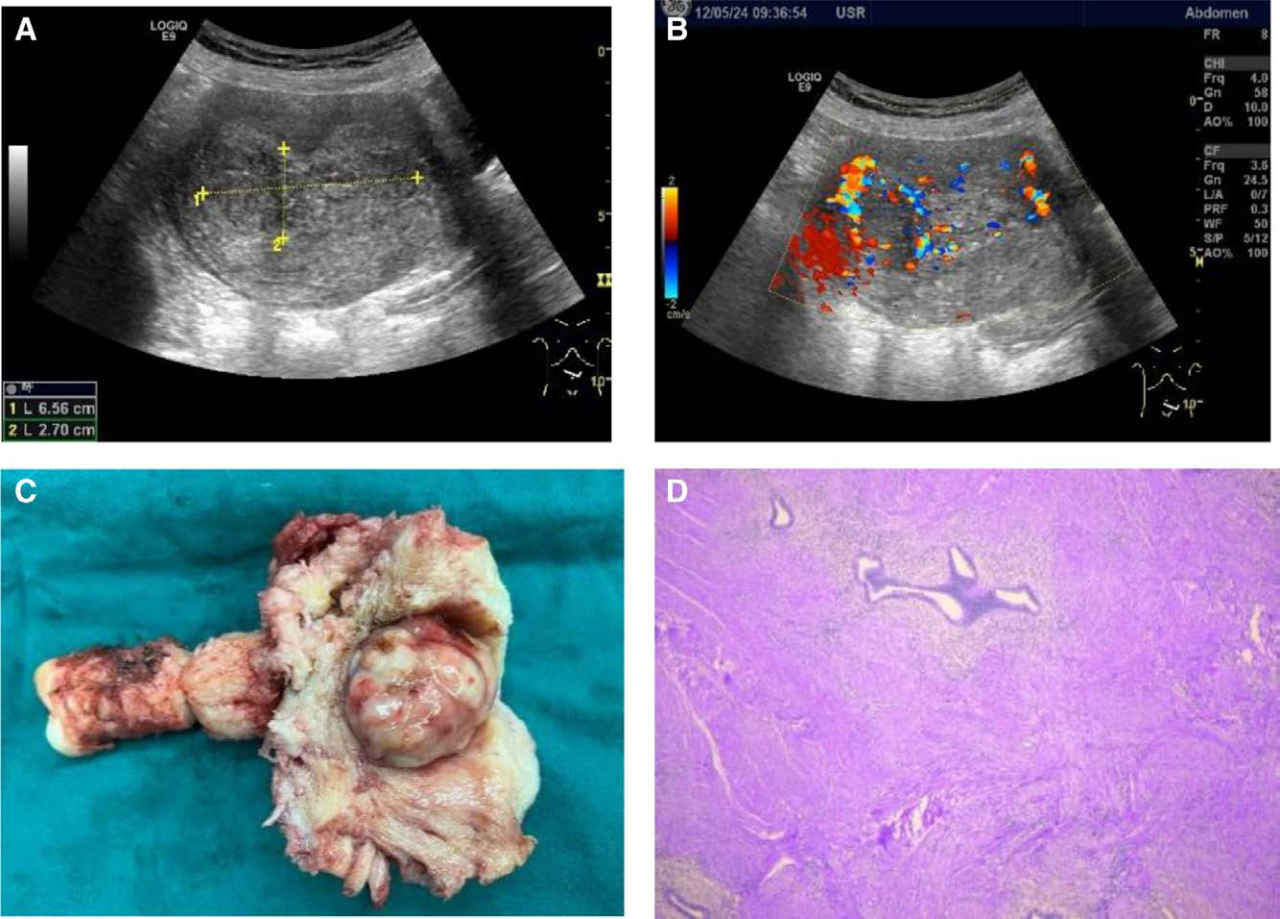
The clinical and pathological features of Patient 2. (A and B) Abdominal and pelvic ultrasonography shows an inhomogeneous mass (approximately 6 × 5 × 3 cm in size) in the uterine cavity, and blood vessels can be seen to penetrate the mass. (C) The macroscopic appearance shows that the mass is light red in color, tough in texture, lobed, and richly vascularized. (D) H&E staining revealed the presence of intravascular smooth muscle tissue that had undergone hemorrhaging and degeneration, exhibiting no discernible heterogeneity.

A gynecological examination revealed that the uterus was enlarged as if the patient were 50 days pregnant, with a hard texture and no obvious tenderness. Pelvic ultrasonography revealed an inhomogeneous echo mass in the uterine cavity, measuring approximately 6.6 × 4.8 × 2.6 cm (Fig. 4). Preoperative CA125 levels were recorded at 80.7 U/mL (normal value: 0–47 U/mL), and the estradiol level on the 14th day of the menstrual cycle was 1068.72 pmol/L. A preliminary diagnosis of adenomyoma or UFs was made and possible IVL was considered.

**Figure 5.**
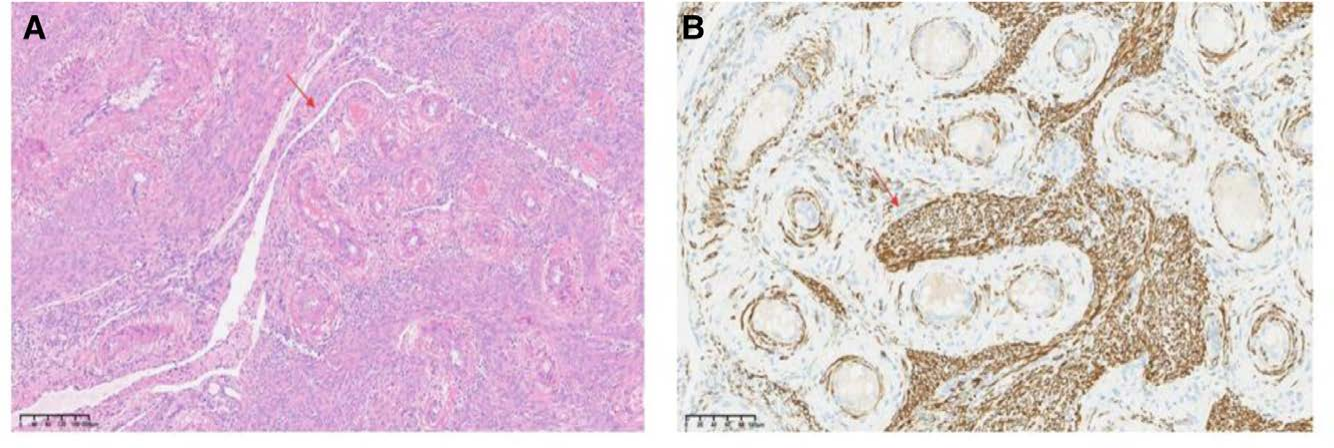
(A) Intravascular tumors filling the venous lumen were stained with hematoxylin and eosin (H&E). The typical spindle cells were arranged in an interlaced pattern, there was no obvious atypia, and the vessel wall had smooth muscle components.(B)The tumor is positive for Desmin marker, confirming the component of smooth muscle in IVL. IVL = intravascular/intravenous leiomyomatosis.

The patient underwent laparoscopic total hysterectomy and bilateral salpingectomy, in which the ovaries were preserved to prevent the effects of estrogen loss. During the operation, the uterus was enlarged to a size comparable to that in the second month of pregnancy, with a smooth surface and no obvious bulges. Following the complete removal of the uterus and bilateral fallopian tubes, an examination revealed a 3 × 4 cm lobulated mass on the anterior uterine wall. The mass was characterized by a soft texture, an abundance of blood vessels on the surface, and a pale red coloration. Microscopic examination revealed the presence of smooth muscle tumor components within blood vessels, indicating local cellular enrichment and intravascular hemorrhage (Fig. 4). Immunohistochemical staining further demonstrated positive expression of P53 (individually), estrogen receptor (ER), desmin, and SMA, along with negative expression of CD10. The Ki-67 index was also <5%, which is consistent with previous reports (Fig. 5). Additionally, the patient exhibited concomitant adenomyosis and endometrial hyperplasia, contributing to a supported diagnosis of IVL.

The patient was discharged 5 days after the operation, with no complications. Subsequent to the surgical intervention, Qiu empirical formula was used, and no subsequent occurrence was identified in the ensuing follow-up evaluations.

## 3. Discussion

IVL manifests predominantly in middle-aged women, exhibiting a protracted and progressive course, and is classified as a tumor with a certain degree of malignancy. The clinical manifestations associated with this disease are nonspecific and variable, contingent on the progression of the mass.^[5]^ Herein, we present 2 cases of IVL that exhibit markedly divergent clinical presentations. Two prevailing theories currently exist regarding the histogenesis of IVL. The 1st suggests that it originates from smooth muscle tissue within the walls of the uterine veins, whereas the 2nd suggests that it originates from uterine leiomyosarcomas that invade extensively into the veins. Immunohistochemistry revealed positivity for desmin and ER, whereas normal myofibroblasts and IVL exhibited strong positivity for desmin and ER. In contrast, smooth muscle cells of the vessel wall were negative for this antibody.^[5,8]^ These findings support the initial theory. Given the medical history of both patients, which included a history of UFs and prior surgical interventions, and the observation that the tumors predominantly traversed the vicinity of the blood vessels associated with the previous fibroid lesions, it is hypothesized that local injury to the uterus and vascular damage may have created the conditions conducive to the development of this disease, thereby influencing the site of tumor growth and the related symptoms they induced.

A clinical staging system for the preoperative assessment of the extent of IVL involvement was proposed by Ma et al^[9]^ in 2016; according to this system, the 2 IVL cases we reported were patients with stage I disease. Because its rare and atypical clinical presentation presents a challenge for accurate diagnosis, the early diagnosis of the 2 patients we reported was made possible by advances in imaging. Preoperative pelvic ultrasonography of the patient in Case 1 revealed a distinct, strip-shaped hypoechoic lesion in the right lateral wall of the uterus, with honeycomb blood flow in the color Doppler flow imaging cross section. This was not identical to the usual ultrasound presentation of a smooth muscle tumor.^[10,11]^ Following refinement of the patient’s enhanced MR image, it was observed that the lesion exhibited isointensity in the T2-weighted imaging signal and equal or mildly elevated intensity in the T2-weighted imaging signal. Multiple tortuous and tangled cords of blood vessels were observed within the tumor and adjacent to the tumor following contrast enhancement, a distinctive imaging feature.^[12,13]^ For the patient in Case 2, the lesion was not diagnosed prior to surgery. This was possible because of the ultrasound, which revealed the lesion, and the color Doppler flow imaging, which demonstrated the lesion’s abundant blood flow and the presence of penetrating blood vessels. This information enabled the lesion to be distinguished from general UFs. In combination with the patient’s medical history, a comprehensive analysis can facilitate early diagnosis and preoperative improvement of relevant examination tests. This allows for the assessment of the patient’s condition and the reduction of surgical risk.

In cases where the tumor is confined to the uterus and pelvis, it can be challenging to accurately diagnose IVL prior to surgery, and the availability of early correlative cues is limited to imaging. It has been posited that IVL is an estrogen-related disease.^[14,15]^ Preoperative estradiol levels in both patients exceeded the mean for women, suggesting that high estradiol levels may serve as an early indicator of risk for IVL in patients with previous UFs. A further challenge in the differential diagnosis of IVL lies in its similarity to other gynecologic diseases, including UFs, uterine malignancies, and adenomyosis, which can complicate the diagnostic process. Intravascular adenomyomatosis is a rare variant of IVL that manifests itself pathologically as an intravascular component of IVL, which also includes endometrioid glandular and stromal components.^[16]^ These components may be associated with each other; however, there is insufficient evidence from relevant studies due to the paucity of cases. Tumor markers can facilitate the preliminary identification of cases. Two patients with mildly elevated preoperative CA125 levels and a history of uterine adenomyosis can be considered concomitant with both conditions, and therefore necessitated our utmost attention.

Surgery is currently recognized as the most effective treatment for IVL; however, the extent of surgery is a subject of controversy. Some scholars suggest performing total hysterectomy and bilateral salpingo-oophorectomy. Preservation of the ovaries and uterus has been shown to increase the likelihood of recurrence.^[5]^ However, a number of scholars have posited that there is no significant correlation between ovarian preservation and recurrence. They contend that complete resection of the lesion is the primary factor in reducing the recurrence rate.^[14,15,17]^ In both cases, the ovaries were preserved, and in Case 1, there was a need for uterine preservation. For this procedure, preoperative deep vein ultrasound and echocardiography, chest and abdominal CT, etc, were required. Following the confirmation of no metastasis of the lesion beyond the uterus, a portion of the uterus, uterine arteries and veins, and the tissues associated with the right round ligament and broad ligament were resected after disconnection of the uterine blood vessels, ensuring complete resection of the lesion. In Case 2, the surgical intervention was more extensive, necessitating the complete removal of both fallopian tubes from the uterus. There is a paucity of evidence to ascertain whether the lymph nodes have been cleared, and the extant literature suggests that IVL rarely metastasizes through the lymphatics. However, the patient’s postoperative immunohistochemistry results suggest that we should be cognizant of the possibility of lymphatic metastasis, and follow-up related to the recurrence of the lesion needs to be emphasized. IVL is regarded as having a quasi-malignant nature, and treatment options for postoperative adjuvant therapy, including endocrine therapy and radiotherapy, are limited and controversial.^[18]^ At present, the clinical application primarily involves estrogen deprivation therapy; however, its therapeutic effect remains to be elucidated. A school of thought among the scholarly community posits that hormone therapy can lead to the shrinkage of lesions. Conversely, an opposing school of thought among the scholarly community contends that gonadotrophin‑releasing hormone agonist does not result in a reduction in the rate of postoperative recurrence in patients with IVL.^[14,15,17]^ The administration of gonadotrophin‑releasing hormone agonist has been observed to hasten the progression of patients into perimenopause and to augment their vulnerability to cardiovascular disease and other concomitant conditions, thereby estrogen deprivation therapy more suitable for younger women. Qiu empirical formula, which has been regarded as having an equivalent estrogen-reducing effect, was administered to 2 patients for an extended period following surgery as an alternative to estrogen deprivation therapy. A subsequent postoperative evaluation revealed a substantial decrease in the estradiol level in both cases. Furthermore, the pelvic ultrasonography in Case 1 demonstrated a substantial reduction in uterine size compared to the preoperative measurement.

This study is subject to certain limitations inherent to case reports. First, this is the 1st instance of 2 cases of IVL being observed at our hospital, resulting in a paucity of experience in diagnosis and clinical management. Second, the limited sample size precluded a systematic investigation of the clinical and pathological characteristics of this rare disease. Finally, the postoperative course of the patients was brief, the patients were observed for a limited period, and the long-term prognosis remains uncertain.

In summary, IVL is a rare, benign muscle cell tumor that manifests without any discernible specificity in the clinical presentations of patients in the early stages of the disease. The diagnosis of this condition necessitates the utilization of imaging procedures and other pertinent diagnostic methods. Treatment entails early surgical complete resection of the lesion. Qiu empirical formula demonstrates application value in long-term postoperative follow-up, given the high recurrence rate, but further research is necessary to substantiate this finding. Clinicians must be cognizant of this particular type of leiomyosarcoma within their clinical practice.

## Author contributions

**Conceptualization:** Yiyang Shi, Jiaxi Wang.

**Data curation:** Chen Zhang.

**Formal analysis:** Xuelu Jiang, Chen Zhang.

**Resources:** Chen Zhang.

**Writing – original draft:** Yiyang Shi.

**Writing – review & editing:** Yiyang Shi, Xuelu Jiang, Chen Zhang.
